# Optimization of Quantum Nuclei Positions with the Adaptive Nuclear-Electronic Orbital Approach

**DOI:** 10.1021/acs.jpca.4c00096

**Published:** 2024-04-15

**Authors:** Lukas Hasecke, Ricardo A. Mata

**Affiliations:** Institute of Physical Chemistry, University of Göttingen, Tammannstrasse 6, 37077 Göttingen, Germany

## Abstract

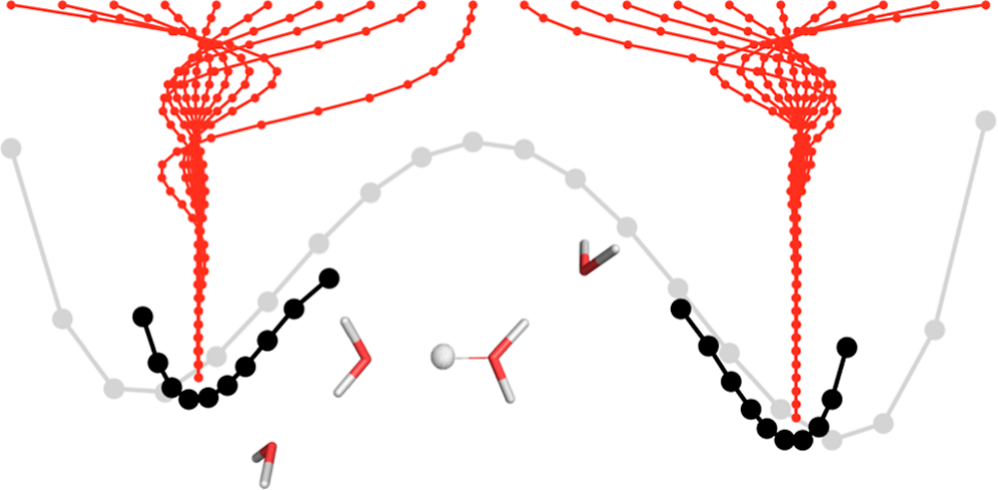

The use of multicomponent methods has become increasingly popular over the last years. Under this framework, nuclei (commonly protons) are treated quantum mechanically on the same footing as the electronic structure problem. Under the use of atomic-centered orbitals, this can lead to some complications as the ideal location of the nuclear basis centers must be optimized. In this contribution, we propose a straightforward approach to determine the position of such centers within the self-consistent cycle of a multicomponent calculation, making use of individual proton charge centroids. We test the method on model systems including the water dimer, a protonated water tetramer, and a porphine system. Comparing to numerical gradient calculations, the adaptive nuclear-electronic orbital (NEO) procedure is able to converge the basis centers to within a few cents of an Ångström and with less than 0.1 kcal/mol differences in absolute energies. This is achieved in one single calculation and with a small added computational effort of up to 80% compared to a regular NEO- self-consistent field run. An example application for the human transketolase proton wire is also provided.

## Introduction

The study of protons in chemical processes can be a challenging task for theory. Movement of protons can strongly couple with electron transfer processes or even be coupled to other proton movements. Adding to these issues are nuclear quantum effects (NQEs) that can vary in magnitude depending on the process. There are several recent and high-profile examples of how proton dynamics are determining the chemical outcome of a system.^1−4^ For example, we have recently shown how a proton wire in the human transketolase system can establish a positive allosteric communication between two active sites more than 25 Å apart.^5,6^ In this study, one observes how proton densities can effectively delocalize and exhibit low transfer barriers, requiring an explicit quantum treatment. Such studies can be quite demanding, requiring quantum molecular dynamics or expensive potential hypersurfaces scans.^5,7,8^ This is linked to some of the most fundamental approximations in quantum chemical calculations, whereby protons are treated as classical particles.

Multicomponent methods allow for the concurrent simulation of quantum electrons and protons. However, when making use of atomic-centered basis functions, care must be taken in placing said functions. In this work, we propose a simple but effective method to dynamically adapt the position of the basis functions to the protonic orbital charge centroids. We compare the results of the approach to numerical optimization results and demonstrate how it effectively simplifies the use of multicomponent methods with stationary geometries.

## Method

In multicomponent methods, one approximates solutions to the Schrödinger equation not only for electrons but also for other particles. The most common application of the method is for the concurrent calculation of quantum protons. The following discussion (and the contents of this paper) is restricted self-consistent field (SCF) methods, meaning Hartree–Fock (HF) and Density Functional Theory (DFT).^9^ In these cases, one describes the total wavefunction as a product of electronic and protonic (both Fermionic) wave functions
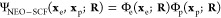
1whereby {**x**_e_} are the electronic coordinates, {**x**_p_} quantum proton coordinates, and {**R**} the positions of classical nuclei. The latter are parameters and not active variables in the problem. The protonic and electronic wave functions will be represented by single Slater determinants, the orbitals being determined by two coupled sets of equations
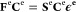
2
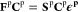
3

The physics of the problem are contained in the Fock matrices (**F**), with the orbitals represented in their basis function space (**C**) and orbital energies (**ϵ**) as solutions. The two systems are coupled by Coulomb operators and, in the case of DFT, by the Coulomb interaction plus electron–proton correlation. The latter term is occasionally disregarded, depending on the implementation and focus of the study.^10,11^ The foundations for these methods were established several years ago by the seminal works of Thomas, Parr, and co-workers.^12,13^

There is, however, one added complication when atom-centered basis sets are used for both electrons and protons. The nuclear positions should be distinguished in two sets, {**R**} = {**r**_**q**_, **r**_**c**_}. The latter correspond to the quantum center **r**_**q**_ and classical nuclei **r**_**c**_ positions. The quantum centers are only placeholders of basis functions, since the respective atom is no longer represented as a point charge in the multicomponent Hamiltonian. Still, it will strongly influence the protonic wave function as it restricts protons to their vicinity. A quantum proton can only exist where nuclear basis functions are provided.

The new potential energy surface (PES) is what is sometimes called in the literature as extended nuclear-electronic orbital (NEO) PES.^14^ This energy *E*(**r**_**q**_, **r**_**c**_) effectively depends on the position of the classical nuclei but is still dependent on where the protonic functions are placed. Technically, one can variationally find the best position for these centers, effectively moving the electronic and protonic basis functions placed at coordinates **r**_**q**_. The PES which respects the condition
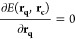
4is commonly designated as standard NEO PES. In this case, the energy *E*(**r**_**c**_) is only a function of the remaining classical atomic centers coordinates. We note, however, that the standard NEO PES is only strictly defined when there is a single minimum for eq 4. Otherwise, one can define several different standard NEO PESs for the same molecular system. In that respect, the extended NEO surface is more generally defined. Both types of PES are defined for the fixed proton basis framework, whereby protonic basis centers are coincident with electronic centers. The difference between the standard and the extended NEO PESs can be reduced if the protonic basis is flexible enough to localize the proton away from the basis center.

**Figure 1:**
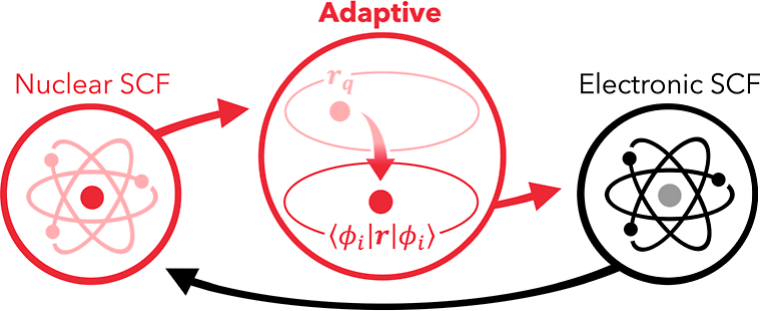
Illustration of the adaptive approach within the stepwise NEO framework. After the quantum nuclear SCF cycles converged, the position of the nuclear basis functions is adjusted to the expectation value of the respective nuclear charge centroids. All quantities which depend on the nuclear positions are updated, and the electronic SCF cycle is started. This scheme is repeated until the NEO calculation and the quantum centers positions converge.

Ideally, it would be best to always work on the standard NEO PES, with optimal **r**_**q**_ values. However, this requires optimizing the respective centers positions. In this contribution, we propose a straightforward extension of the NEO method, whereby proton positions can be determined during the SCF cycles, with little total added cost. The procedure consists of computing the charge centroids of the individual protons and updating the **r**_**q**_ positions to the latter values. These are directly obtained as the expectation values of each nuclear orbital ϕ_*i*_ for the Cartesian coordinates’ operators ⟨ϕ_*i*_|**r**|ϕ_*i*_⟩ (**r** = {*x*, *y*, *z*}). Each orbital ϕ_*i*_ is mapped to an atomic center *i*. An update step is carried out at each SCF cycle. A schematic of the procedure is provided in Figure 1. It should be noted that our NEO-HF implementation performs the SCF cycles stepwise, first solving the nuclear Roothaan and Hall equations before performing a full cycle for the electrons and repeating the process.^15^ The nuclear positions are updated after each nuclear SCF cycle. From the calculations we have carried out so far, the change in position does not affect the SCF convergence significantly.

The method could be straightforwardly applied in combination with quantum crystallography in qualitatively assessing NQEs. The NEO framework can theoretically provide such results within a fraction of the cost of other methods.^8^ This is a rather unique case in quantum chemical treatments, where instead of a nuclear gradient, one follows an expectation value to achieve a (partial) structure optimization. Comparison to gradient-based optimizations shows close agreement at a fraction of the computational cost, demonstrating that the standard NEO PES can be effectively approximated.

There are several different multicomponent variants available (nuclear orbital molecular orbital (NOMO),^16^ dynamical extended molecular orbital (DEMO),^17^ multicomponent molecular orbital (MCMO),^18^ and electronic and nuclear molecular orbitals (ENMO)^19^ to name a few), but we will be making use of our local density fitting nuclear electronic orbital restricted Hartree–Fock implementation (LDF-NEO-RHF) for this work.^20^ The method is only qualitative as it tends to overlocalize the protonic densities. The energies obtained are also to be taken with caution, since no electron–proton correlation is included.^21^ However, for the purpose of this paper, one requires only an SCF procedure, with the accuracy playing no decisive role. The adaptive approach discussed in this contribution is not limited to NEO-HF nor hydrogen atoms but can be applied to other multicomponent HF and DFT ansatzes and heavier atoms as well.^22^ Over the years, the NEO ansatz and beyond have been applied not only to a myriad of wave function methodologies^9,23−27^ but also to DFT (NEO-DFT).^28−31^ The protonic charge centroid is also used in the constrained NEO approach^32^ from Yang and co-workers, albeit for a different purpose. The proton is forcibly constrained to the center of functions, which allows for a well-defined PES and molecular dynamics simulations.^10^ The focus of this work, nonetheless, is different. One aims to determine the optimal positions for the basis centers within the SCF cycle.

## Computational Details

The adaptive approach is straightforward to implement in multicomponent methods. At the end of each nuclear SCF cycle, charge centroids are computed for all nuclear centers. This is performed with the corresponding operator in a Cartesian space ⟨ϕ_*i*_|**r**|ϕ_*i*_⟩, with the nuclear orbital ϕ_*i*_ being mapped to a nuclear center *i*. Afterward, the quantities which are dependent on the nuclear coordinates need to be updated. In the case of our integral-direct implementation in Molpro, this update is only necessary for the one-particle integrals and therefore computationally inexpensive.

In our implementation, the adaptive procedure starts with the first converged nuclear wave function in the multicomponent SCF cycle. As convergence criteria we employ the energy difference, the density difference, the gradient, and the difference in the position of the centroids. For all calculations, a threshold of 10^–8^ au is used for the energy difference within the nuclear and electronic SCF cycles and the difference in the density between iterations and the gradient. A threshold of 10^–6^ Hartree for the minimal required energy difference in the NEO-RHF iterations was set. For the convergence of the nuclear positions, a threshold of 10^–5^ Bohr was set. All criteria must be fulfilled for the convergence of the adaptive procedure. We employed the cc-pVTZ and cc-pV5Z basis sets with the cc-pVTZ-JKFIT and cc-pV5Z-JKFIT basis sets for density fitting, respectively.^33,34^ For the nuclear basis, we employ the PB4-D, PB4-F2, and PB5-G basis sets.^35^dditionally, for the density fitting of the coupling contribution, the 10s10p10d10f for the PB4-D and PB4-F2 basis sets and 10s10p10d10f10g for the PB5-G basis set are employed. The even-tempered sets have exponents ranging from  to 64. In order to accelerate the electronic and nuclear SCF convergence, we employ the direct inversion in the iterative subspace (DIIS) starting after the first iteration with 10 Fock matrices as basis to extrapolate during the subiterations.^36,37^ The optimization with numerical gradients was carried out with the Molpro default procedure for the LDF-NEO-RHF program.^20,38^ The molecular structures and the contour representation of the nuclear density were created with the PyMOL 2.5.2 program.^39^ The structure of the water dimer was obtained from the Computational Chemistry Comparison and Benchmark DataBase optimized at the CCSD(T) level with the aug-cc-pVQZ basis set.^40^ The structures of the protonated water tetramer and the porphine molecule were obtained from Dickinson et al.,^41^ and the cluster model of the transketolase system was generated based on the structure from Dai et al.^5^

## Results

With the aim to benchmark the robustness and performance of the adaptive nuclear-electronic orbital approach, we depicted three representative test systems. These systems not only scale with size and therefore with the computational effort but also exhibit increasing amount of NQEs and complexity in the underlying PES. The first and simplest system is the water dimer with a single-well potential for the central hydrogen. A more complex PES is given for the trans-Zundel protonated water tetramer, where a double-well potential with two distinct energy levels is observed for the central proton. The most challenging system, in terms of complexity, is the porphine molecule. It exhibits two energetically degenerate double-well potentials for the double hydrogen transfer.^41,42^

**Figure 2:**
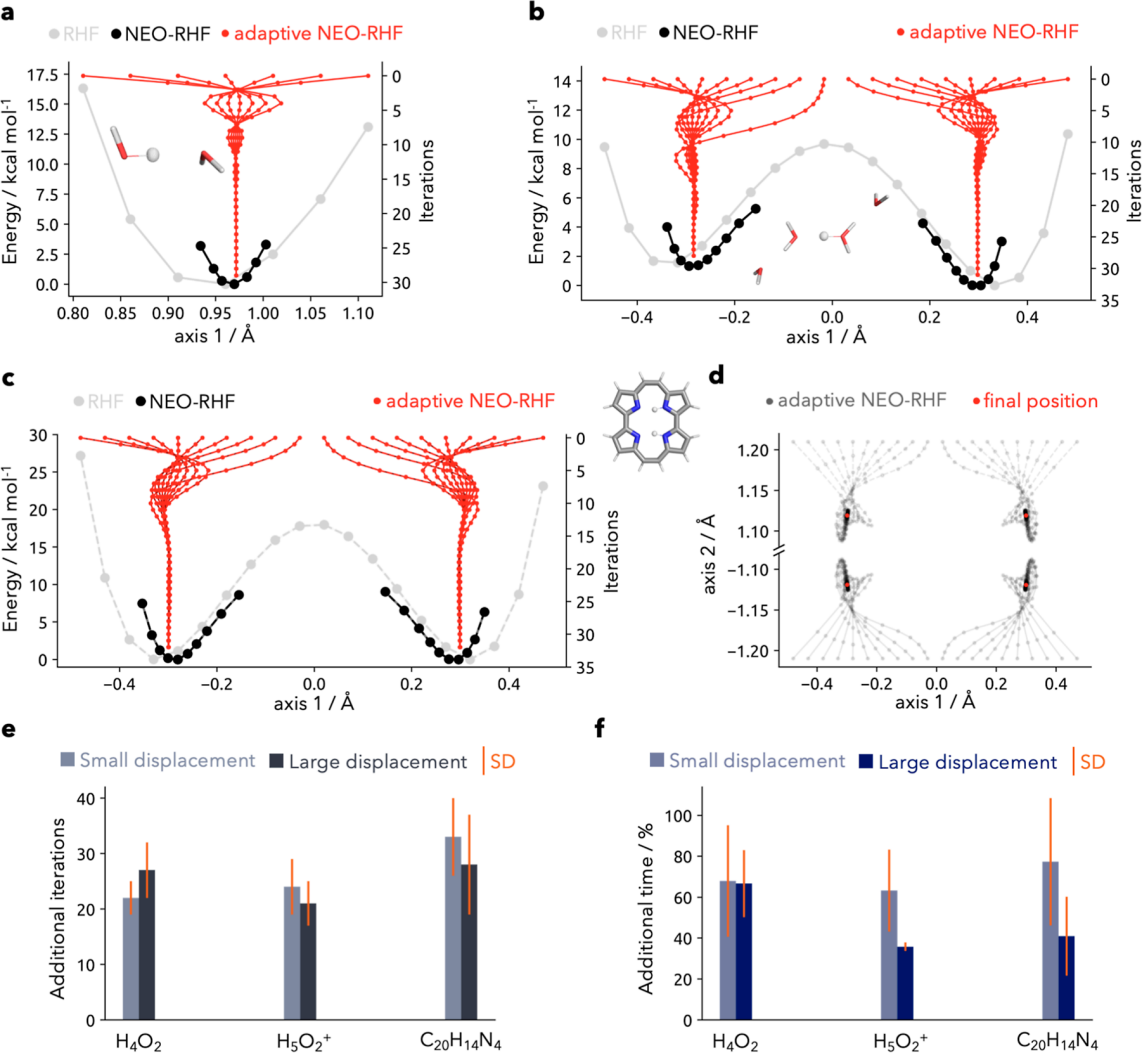
(a–c) PES of the water dimer, protonated water tetramer, and porphine system (nuclear density shown at a 0.01 σ contour level) computed with RHF (light gray) and NEO-RHF (black). The convergence of the adaptive procedure with respect to the principal coordinate axis is shown in red. Different initial structures have been used. The nuclear coordinates for the NEO-RHF and adaptive scheme are given as the expectation value. (d) Nuclear coordinates update for the porphine system along both axes. The values for four distinct minima are marked in red. (e) Additional iterations for the trial systems needed by the adaptive procedure compared to the regular NEO-RHF for a small displacement (the minimum structure and the two closest displacements) and larger displacement (maximum displacements away from the minimum structure along both directions) including the standard deviation. (f) Additional time of the adaptive procedure for the trial systems at a small and large displacement with respect to a standard NEO-RHF energy calculation.

The respective PESs are listed in Figure 2a–c. When comparing the results obtained from regular electronic structure HF and the results from multicomponent NEO-HF it is apparent that the minima differ. In the case of the trans-Zundel tetramer (Figure 2b), the minima move closer together by about 0.1–0.2 Å. These results illustrate how NEO energies can be influenced by the choice of the structure. If the atomic basis center positions (**r**_**q**_) are not optimally placed, the nuclear density will be polarized. This will commonly happen when a Born–Oppenheimer minimum is used, potentially resulting in artifacts. First and foremost, there is an energy penalty incurred when the nuclear charge centroid significantly differs from the position of the assigned atomic center.

We now turn to the adaptive NEO results, where we update the atomic center position to that of the charge centroid. In the same figures, we show the positions of the quantum nuclei along the NEO SCF iterations. There are several red lines, each depicting a different adaptive NEO calculation with different starting positions on the PES. They all ultimately converge to the closest local minimum of the NEO PES. Since the centroid movement is not restricted to the original slice of the PES also some movement along the remaining axes is observed. This is shown in Figure 2d for the porphine system, which illustrates the four distinct minima in the PES. Furthermore, in Figure S1a–c, the water dimer and trans-Zundel tetramer movement along the other axes is displayed. In addition, one can see in Figure 2b,c that some structures were close to the actual transition state, since only a minor change of the position is observed during the first iterations.

Overall, the positional change with the adaptive approach shows a distinct ripple pattern, which is also reflected in the energy convergence compared to the regular NEO-RHF. This is shown in Figures S1d–i. For the study of the energy convergence, the minimum structure as well as a displaced structure, where the nuclear basis set center is moved toward the closest classical nucleus, are examined. In the case of the regular multicomponent calculation (whereby all atomic centers are kept fixed), one observes a smooth energetic convergence for all components. During the iterations, the centroid is slowly shifting away from the classical nucleus, which minimizes the nuclear and electronic energies. However, the coupling energy between both is decreasing since, by moving away from the atomic center, the interaction with the surrounding electronic density is lowered. This behavior is more pronounced for the displaced structure, as expected. However, also for the minimum structure, this behavior is observed and is in agreement with the shift of the PES minima for the regular and multicomponent calculation shown in Figure 2a–c. In the case of the adaptive approach, one can clearly see the quantum nuclei moving away from the classical nuclei, lowering the nuclear energy and decreasing the coupling energy. Some oscillatory behavior is observed until the optimal position (overall energetic minimum) is found. Damping functions can be used to avoid these behaviors (Figure S2) and possibly accelerate the convergence for complex systems. However, at this stage, we see little need for this algorithmic change. The convergence pattern is still robust for all systems tested.

For a quantitative assessment of the computational costs, we show the additional iterations which the adaptive procedure needs in comparison to a regular multicomponent calculation in Figure 2e. The overall trend is almost unaffected by the individual system with a mean between 21 and 31 additional iterations. Moreover, the difference of the additional iterations between the small displacements and large displacements is very small. In terms of computational time, these additional iterations lead to only a mean increase of 36–77% (Figure 2f). For the trans-Zundel tetramer and the porphine, the larger displacement even shows a lower additional time. This is due to difficulties in converging the NEO wave function when the basis functions are inadequately placed. One should also note that the additional time for adaptive NEO close to the minimum structure is not found to be drastically size-dependent. Overall, the mean additional time stays below 100%. This means that the time it takes to find an optimal position for the quantum nuclei is less than two full NEO calculations. The efficiency is even more impressive when compared to gradient-based optimizations, which require multiple NEO calculations. Test calculations for the single-quantum proton systems used in this work show that 5–10 iterations are required till convergence. Albeit the SCF cycles after the first calculation are faster (due to better starting orbitals), one requires a full gradient (electronic and nuclear terms), which is still computationally more expensive than a single point run.

**Figure 3:**
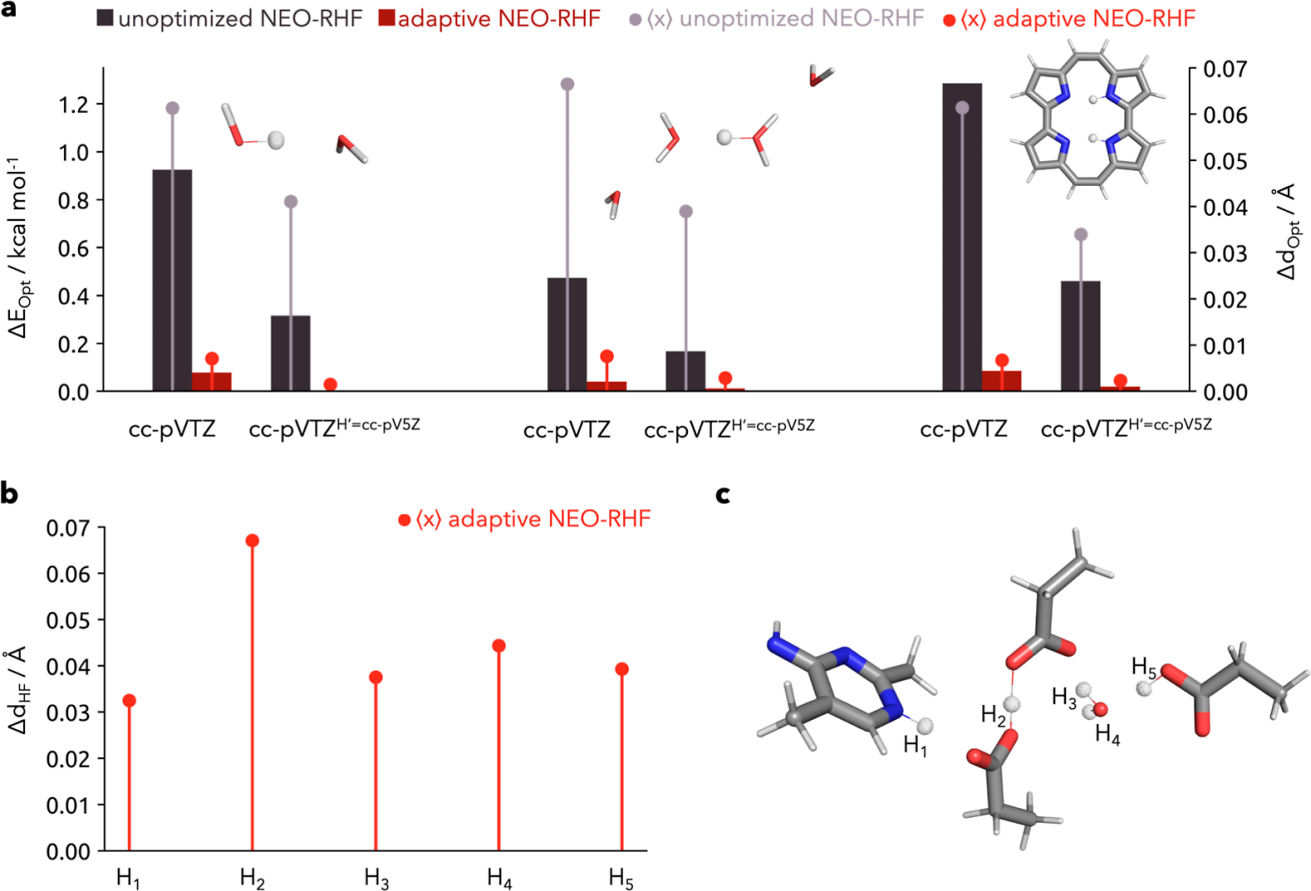
(a) Influence of the electronic basis set on the centroid shift and energy difference of the unoptimized NEO-RHF and adaptive procedure compared to results from numerical gradient optimizations for the trial systems, starting from a slightly displaced structure (0.05 Å apart from the minimum structure in the PES). By giving the electrons surrounding the quantum nuclei the possibility to delocalize further within a larger basis set (cc-pV5Z instead of cc-pVTZ), the centroid can move further toward the minimum, and the energetic difference is subsequently lower. This effect is clearly more pronounced for the regular NEO computation, but the adaptive procedure is also slightly affected. (b) Difference of the nuclear positions obtained from an optimization with regular HF and the adaptive NEO-RHF for a transketolase cluster model. (c) Visualization of the transketolase cluster model with the nuclear density shown at a 0.01 σ contour level.

In order to benchmark the accuracy of the obtained minima from the adaptive approach and elucidate the limitations of the nuclear centroid shift in dependence of basis sets, we compare these to results from numerical gradients. The difference in energy and position for the trial systems in dependence of the electronic basis set is shown in Figure 3a. Moreover, the influence of the nuclear basis set on the accuracy and the shift is shown in Figure S3. It is obvious that by giving the electrons surrounding the quantum nuclei more flexibility by the use of a larger basis set (cc-pV5Z), the centroid can move further toward the minimum. With smaller electronic basis sets, the electron density remains strictly at the functions center, restricting the nuclear density as well (independent of the protonic basis chosen). This effect is clearly more pronounced for the unoptimized NEO computation, but the adaptive procedure is also slightly affected. Moreover, the size of the nuclear basis set is rather important for the delocalization of the quantum nuclei (see Figure S3). One then observes a larger centroid shift for the unoptimized NEO-RHF computation. However, the adaptive approach is already for the smaller basis set in great agreement with the results of the numerical gradient-based optimization.

The difference in the positions is only between 0.007 and 0.008 Å and energetically between 0.04 and 0.09 kcal mol^–1^. Moreover, with the larger electronic basis set and the identical nuclear basis set, the difference in the position decreases to 0.001–0.003 Å and energetically to 0.001–0.02 kcal mol^–1^. Therefore, the adaptive approach is a fast and accurate alternative to gradient-based optimizations in multicomponent methods. Moreover, the adaptive procedure is numerically stable within the set thresholds, resulting in the same position and energy for every initial structure associated with the obtained local minimum. The standard deviations for the water dimer are 0.0004 kcal mol^–1^ and 4 × 10^–5^ Å, for the protonated water tetramer 0.0003 kcal mol^–1^ and 3 × 10^–5^ Å, and for the porphine system 0.0004 kcal mol^–1^ and 6 × 10^–6^ Å.

The efficiency of the adaptive approach allows a routine optimization of quantum nuclei for large systems especially relevant for quantum crystallography, the development of new materials, in silico drug design, and catalysis.^2,5,43−47^ In Figure 3b,c, we show the quantitative difference in the nuclear positions for a transketolase cluster model. In general, the difference of the positions with and without the inclusion of NQEs ranges from 0.03 to 0.07 Å. The proton in the crystallographically identified low-barrier hydrogen bond (H_2_) clearly shows the highest difference. A classical treatment of the proton underestimates how much the proton is shared among the two residues. Especially in these cases, an accurate description of the nuclear positions is essential to correctly elucidate chemical reactivity.^2,5,7^

## Conclusions

In this contribution, we present a cost-effective method to refine proton positions under a quantum treatment. The approach is generally applicable to SCF multicomponent calculations and could prove a useful tool for the study of NQEs in amorphous and crystalline materials. We present a small cluster model example of the proton wire identified in the human transketolase system. One of the main advantages of the method is its simplicity, allowing for seamless integration in the SCF cycles. In this way, the quantum proton positions can be refined together with the wave function/density optimization at an extremely low cost. Through the use of adaptive NEO, one can avoid biases in energy calculations as the basis function centers are adapted to best fit the converged proton and electronic structure. One further advantage of this approach is that it can be straightforwardly extended to correlated wave function methods. For example, in NEO-MP2 or other multicomponent correlated wave function approaches, one can compute the correlation density from the respective amplitudes and extract a total nuclear density, which can be used to further refine the proton positions.^48,49^

## Data Availability

All structural information together with the corresponding energies are available free of charge on GRO.data (10.25625/CJKT1W).

## Supporting Information Available

Positional change of the nuclear coordinates during the adaptive procedure for the water dimer and the protonated water tetramer, energetic change of the electronic, nuclear, and coupling energies for regular NEO-RHF and the adaptive procedure for all trial systems, convergence pattern with different damping schemes for the water dimer, and results shown in Figure 3a (PDF)
