# Supplementary material for: Optimization of Quantum Nuclei Positions with the Adaptive Nuclear-Electronic Orbital Approach
Source: J Phys Chem A. 2024 Apr 15;128(16):3205–11. doi: 10.1021/acs.jpca.4c00096 (PMC11056972; doi:10.1021/acs.jpca.4c00096)
Supplement: Supplementary file 1 — jp4c00096_si_001.pdf [file jp4c00096_si_001.pdf]

Optimization of quantum nuclei positions with the adaptive Nuclear-Electronic Orbital approach

Lukas Hasecke* and Ricardo A. Mata*

Institute of Physical Chemistry, University of Göttingen, Tammannstrasse 6, 37077, Göttingen, Germany

E-mail: lhaseck@gwdg.de; rmata@gwdg.de

Supporting Information

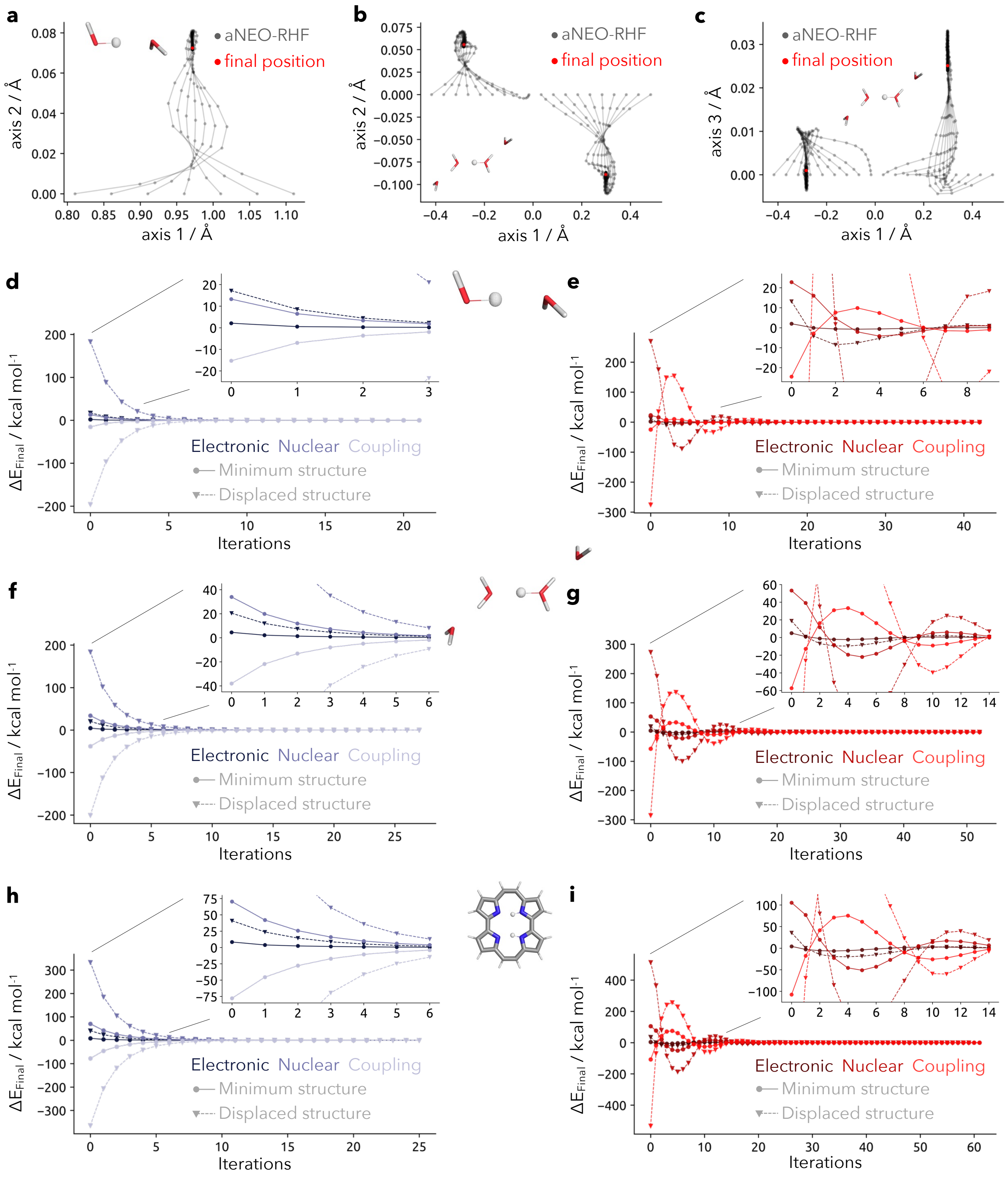

Figure S 1: a-c) Positional change of the nuclear coordinates during the adaptive procedure of the water dimer and protonated water tetramer along the other axes. It should be noted that the symmetry of the water dimer and the porphine system (Fig. 2d) is also reflected in the adaptive procedure, where the nuclear centroid only moves along two directions. d-i) Change of the characteristic energy contributions (electronic, nuclear and coupling energies) for regular NEO-RHF (left side) and adaptive NEO-RHF (right side) for two structures of each system. One structure which is the closest to the minimum in the PES and one displaced structure, where the quantum nuclei are located close to the classical nuclei in the beginning.

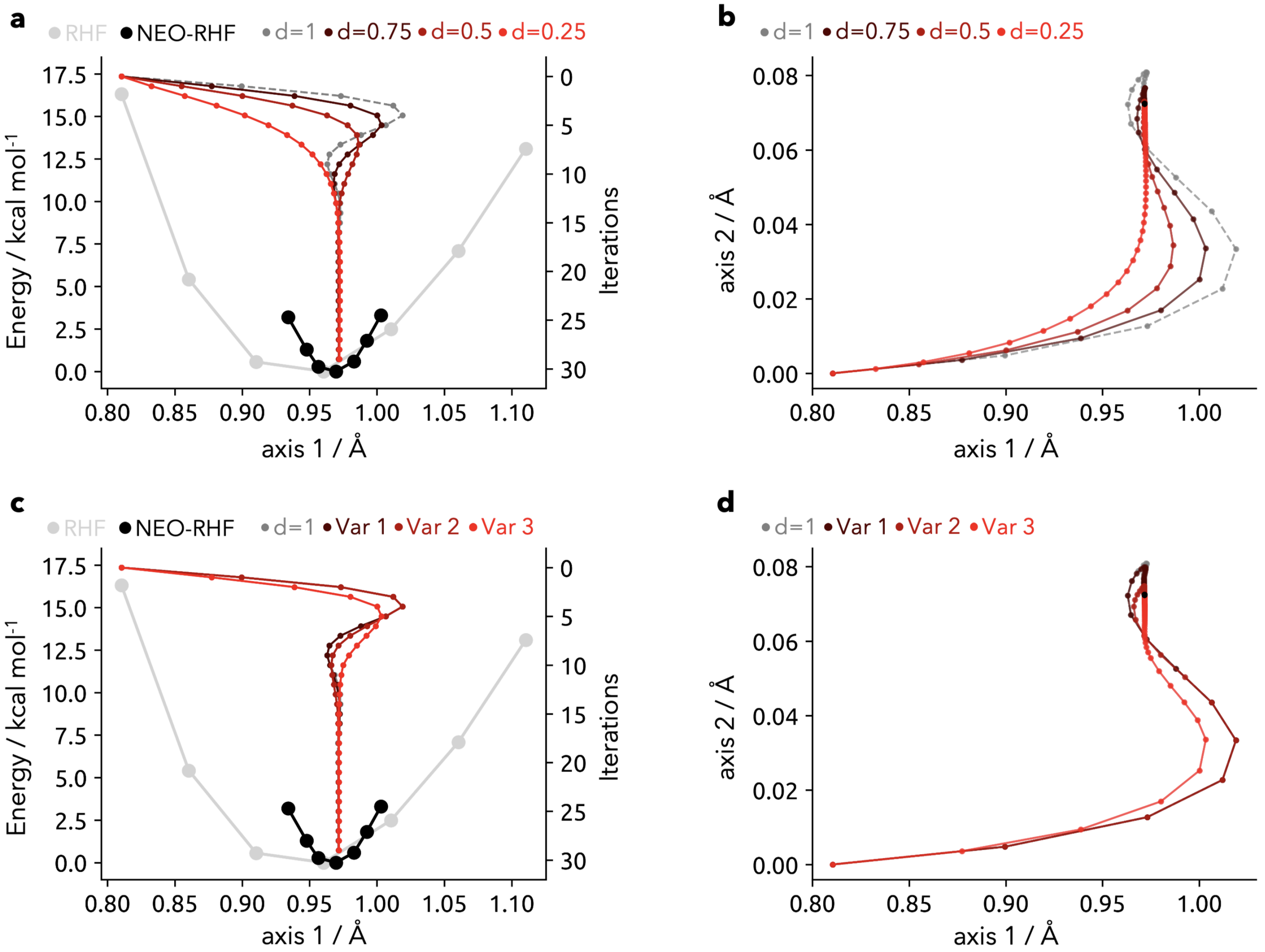

Figure S 2: Convergence pattern of the adaptive NEO-RHF for the water dimer with different damping approaches. a-b) Static damping of the nuclear centroid. During the iterations the movement of the nuclear centroid is restricted by the respective damping factor as $r_{i+1} = r_i + d\Delta r_i$ with $\Delta r_i = r_{i+1} - r_i$. As shown low damping factors reduce the observed ripple pattern of the adaptive approach. However, this results in an increased number of iterations until convergence is archived. c-d) Increasing damping approach in different variants. Variant 1 uses for the first 10 iterations no damping and starts then for 5 iterations with a damping factor of 0.75 which is decreased after 5 iterations to 0.5 and after additional 5 iterations to 0.25 until convergence is reached. Variant 2 starts with the same damping procedure at the 5th iteration and Variant 3 at the first iteration. With this procedure only the later oscillations of the nuclei are reduced.

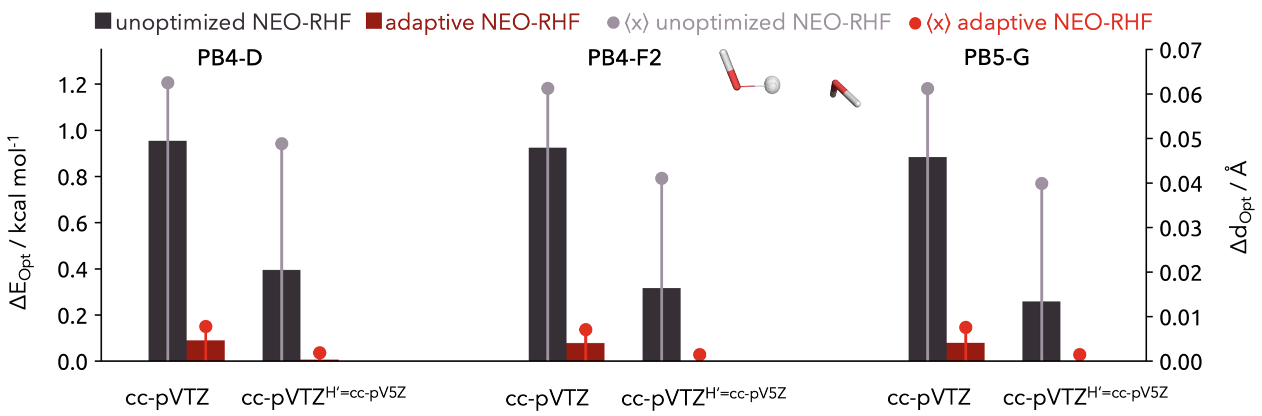

Figure S3: Influence of the nuclear and electronic basis set on the centroid shift and energy difference of the unoptimized NEO-RHF and adaptive procedure compared to results from numerical gradient optimizations for the water dimer, starting from a slightly displaced structure (0.05 Å apart from the minimum structure in the PES).

Table S1: Influence of the electronic basis set on the centroid shift and energy difference of the unoptimized NEO-RHF and adaptive procedure compared to results from numerical gradient optimizations for the trial systems (as graphically shown in Fig. 3).

unoptimized NEO-RHF			
system	water dimer	protonated water tetramer	porphine
basis	PB4-F2/cc-pVTZ		
ΔE / kcal mol^{-1}	0.9251077	0.4732322	1.2855333
Δd / Å	0.0612721	0.0664920	0.0613645
basis	PB4-F2/cc-pVTZ/H1(-H2)=cc-pV5Z		
ΔE / kcal mol^{-1}	0.3157445	0.1670308	0.4605854
Δd / Å	0.0410836	0.0389802	0.0339700
adaptive NEO-RHF			
basis	PB4-F2/cc-pVTZ		
ΔE / kcal mol^{-1}	0.0787542	0.0400433	0.0850805
Δd / Å	0.0070898	0.0075777	0.0067592
basis	PB4-F2/cc-pVTZ/H1(-H2)=cc-pV5Z		
ΔE / kcal mol^{-1}	0.0018748	0.0117000	0.0200652
Δd / Å	0.0014912	0.0028797	0.0023533
